# The Popeye Domain Containing Genes and cAMP Signaling

**DOI:** 10.3390/jcdd1010121

**Published:** 2014-05-21

**Authors:** Thomas Brand, Kar Lai Poon, Subreena Simrick, Roland F.R. Schindler

**Affiliations:** Harefield Heart Science Centre, National Heart and Lung Institute (NHLI), Imperial College London, Hill End Road, Harefield UB96JH, UK

**Keywords:** Popeye domain containing genes, cAMP, phosphate binding cassette, cAMP-binding proteins, pacemaking, cardiac arrhythmia, fight-or-flight response, sinus node, cardiac conduction system

## Abstract

3'-5'-cyclic adenosine monophosphate (cAMP) is a second messenger, which plays an important role in the heart. It is generated in response to activation of G-protein-coupled receptors (GPCRs). Initially, it was thought that protein kinase A (PKA) exclusively mediates cAMP-induced cellular responses such as an increase in cardiac contractility, relaxation, and heart rate. With the identification of the exchange factor directly activated by cAMP (EPAC) and hyperpolarizing cyclic nucleotide-gated (HCN) channels as cAMP effector proteins it became clear that a protein network is involved in cAMP signaling. The Popeye domain containing (Popdc) genes encode yet another family of cAMP-binding proteins, which are prominently expressed in the heart. Loss-of-function mutations in mice are associated with cardiac arrhythmia and impaired skeletal muscle regeneration. Interestingly, the cardiac phenotype, which is present in both, *Popdc1* and *Popdc2* null mutants, is characterized by a stress-induced sinus bradycardia, suggesting that Popdc proteins participate in cAMP signaling in the sinuatrial node. The identification of the two-pore channel TREK-1 and Caveolin 3 as Popdc-interacting proteins represents a first step into understanding the mechanisms of heart rate modulation triggered by Popdc proteins.

## 1 Introduction

**Figure 1:**
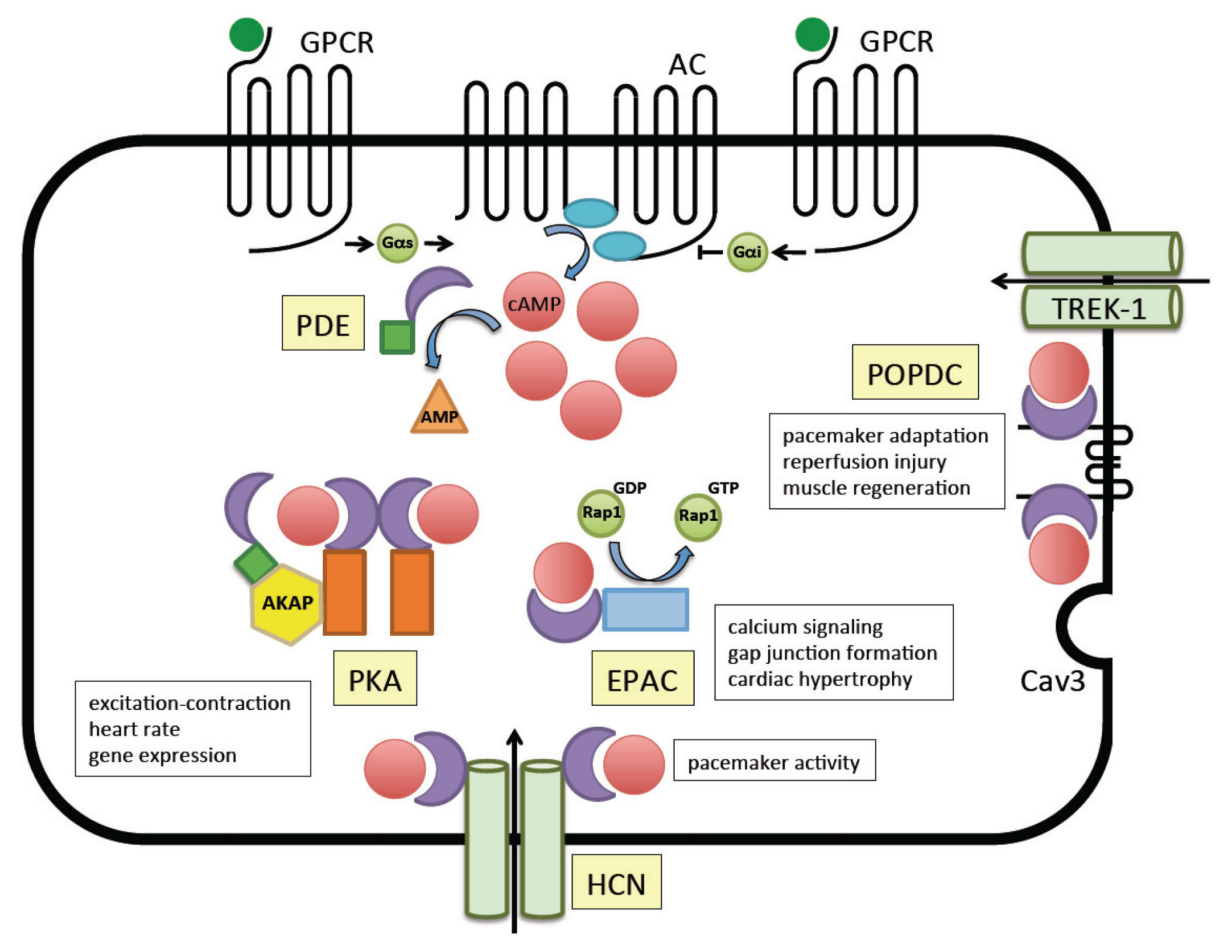
Schematic overview of the elements of the cAMP signal transduction pathway in cardiac myocytes. G-protein coupled receptors (GPCRs) such as the β-adrenergic receptor activate Gαs causing a stimulation of cAMP production by adenylate cyclase (AC). Other GPCRs such as the muscarinic acetylcholine receptor couple with GαI causing an inhibition of AC. cAMP production is highly controlled in a spatiotemporal manner. Main control of compartmentalization is through the rapid degradation of cAMP by phosphodiesterases (PDE) which hydrolyzes cAMP to adenosine monophosphate (AMP). PDE are often present in close neighborhood with protein kinase A (PKA) through association with A kinase anchor proteins (AKAP) proteins, which are responsible for localizing PKA to different compartments in the cell. Other cAMP-binding domain (CNBD) containing proteins are the hyperpolarization-activated cyclic nucleotide-gated (HCN) channels, which play a major player in cardiac pacemaking. The exchange factor directly activated by cAMP (EPAC) is a guanine nucleotide exchange factor, which modulates Ras related protein 1 (Rap1), a small GTP-binding protein. The Popeye domain containing (Popdc) proteins represent yet another class of cAMP-binding proteins, which play an important role in cardiac pacemaking. Popdc proteins interact with the two-pore potassium ion channel TWIK related K-1 (TREK-1) and Caveolin 3 (Cav-3) [4,19]. For each cAMP effector protein a few examples of functions in striated muscle tissue are given in the white boxes adjacent to each molecule. It becomes apparent that the cAMP-binding proteins display significant functional overlap. It should also be mentioned that different cardiac muscle cells differ in the level of expression of some the depicted effector proteins. For example, the sinuatrial (SAN) and atrioventricular (AVN) nodes express higher levels of HCN1 and HCN4 and Popdc1 and Popdc2 than atrial and ventricular muscle cells.

The second messenger cyclic adenosine 3', 5'; monophosphate (cAMP) plays a prominent role in cardiac physiology and signal transduction. cAMP is produced by adenylate cyclase (AC). There are ten different AC, nine of which are membrane-bound (AC1-9) and activated by G protein-coupled receptors (GPCRs) or by Ca^2+^ (Figure 1) [1]. The tenth AC is soluble (sAC) and is regulated by bicarbonate [2]. AC5 and -6 are the major isoforms present in cardiac myocytes and are coupled for example to adrenergic and cholinergic receptors through stimulatory and inhibitory Gα-subunits. Once activated, AC produces a large amount of cAMP, which passively diffuses in cells. cAMP is rapidly degraded by phosphodiesterases, which thereby ensure that cAMP diffusion is limited and usually confined to a small area around the site of production. There are three cAMP effector protein classes known: protein kinase A (PKA), exchange factor directly activated by cAMP (EPAC), and cyclic nucleotide-activated ion channels, which includes cyclic nucleotide–gated (CNG) channels, mainly expressed in photoreceptors and olfactory sensory neurons, and hyperpolarization-activated cyclic nucleotide-gated (HCN) channels, which are expressed in heart and brain [3]. We recently discovered a fourth class of cAMP-binding proteins, the Popeye domain containing proteins (Popdc), which are the main focus of this review article [4–7]. A fifth potential cAMP-binding protein, Cris was recently identified, which however is exclusively expressed in sperm precursor cells [8].

## 2 The Popeye Domain Containing Proteins

The Popeye domain containing (Popdc) genes were discovered 15 years ago after screening cardiac cDNA libraries with the goal to identify genes with a cardiac-enriched expression pattern [9,10]. Immunohistochemistry, in situ hybridization, LacZ staining of knock-in reporter genes and RT-PCR established an exclusive expression in cardiac myocytes [4,10–13]. This expression pattern is evolutionary conserved [14–16].

There are three Popdc genes in vertebrates, *Popdc1* (also known as *Bves*), *Popdc2* and *Popdc3* [10]. Popdc genes encode small membrane-localized proteins, which are between 290–360 amino acids long. They have three transmembrane domains and in the cytoplasmic part of the protein, a highly conserved Popeye domain is present [10]. Popdc proteins are unique and do not resemble any other non-Popdc protein. The proteins with the highest sequence similarity are the bacterial cAMP regulator proteins (CRP) [6], which encode transcription factors that modulate gene expression in response to metabolic changes [17]. While a transcriptional regulatory role has yet to be demonstrated for Popdc proteins, both Popdc1 and Popdc2 have been found in the nucleus of striated muscle cells in addition to their presence at the plasma membrane [18].

The Popeye domain displays significant structural homology to cAMP-binding proteins [4]. A homology model was devised and is based on structural information of CRP and PKA. This model revealed that a large fraction of invariant amino acids cluster around the putative cAMP-binding domain suggesting their involvement in nucleotide binding [4,6,7]. Indeed, for a number of these conserved residues, mutagenesis revealed an essential function in cAMP-binding. In particular, the aspartate residues D200 in Popdc1 and D184 in Popdc2 appear to be essential for nucleotide binding [4,6,7]. Surprisingly the protein sequence of the phosphate-binding cassette (PBC) is unique and different from that of HCN4, EPAC, or PKA [6,7]. This suggests an independent evolution of the PBC of Popdc proteins. Surprisingly, the PBC of Popdc proteins found in basic metazoans such as cniderians (*Hydra*) is almost identical to the PBC found in Popdc proteins from higher vertebrates [6] demonstrating strong evolutionary pressure to retain these sequences and providing further evidence for their functional importance. Affinity measurements revealed that Popdc proteins bind cyclic nucleotides with high affinity in the physiologically relevant range [4].

## 3 Loss-of-Function Mutations of Popeye Domain Containing Genes are Causing Cardiac Arrhythmia

All three members of the Popdc family are abundantly expressed in cardiac and skeletal muscle. Loss-of-function experiments in zebrafish using morpholino-mediated knockdown caused defective development of skeletal muscle in the head and trunk [14]. While muscle development is apparently normal in mice, there is a retarded ability of skeletal muscle regeneration [11]. In the zebrafish heart of *popdc2* morphants, a severe atrioventricular block is observed [14]. Interestingly, a cardiac arrhythmia phenotype is also found in mutant mice. In both *Popdc1* and *Popdc2* null mutants in mice, a bradyarrhythmia phenotype has been described [4]. While at baseline no difference is present between mutant and wildtype, subjecting null mutants to physical or mental stress caused sinus pauses, which randomly occurred but were strictly stress-dependent [4]. Moreover, for both *Popdc1* and -*2* mutants, phenotype development is age-dependent. Young mutants (3 months old) display a normal chronotropic competence, while middle-aged mutant mice (6–8 months old) are severely bradycardic [4]. The age-dependency of the stress-induced bradycardia phenotype in these mouse mutants is reminiscent of sinus node dysfunction (SND) in man. SND is a leading cause for pacemaker implantation and patients with SND, which is prevalent in the elderly, have difficulties to adapt the beating frequency of their heart to the physiological demands [20]. It has therefore been hypothesized that some SND patients may have abnormal Popdc gene expression or function [4]. Although such data are presently unavailable, in failing hearts, the expression levels of *POPDC1* and *POPDC3* are reduced, however, the degree of down-regulation varies between patients [21]. Reduced levels of *POPDC1* and *POPDC3* might represent risk factors for the development of sudden cardiac death, atrial fibrillation and other conduction disorders, which are prevalent among heart failure patients.

## 4 Protein-Protein Interactions of Popdc Proteins

Our understanding of the molecular function of Popdc proteins is currently limited due to the fact that we do not know the protein interaction partners of Popdc proteins. However, two relevant interacting proteins have now been identified.

### 4.1 TREK-1

It has been reported that Popdc proteins interact with the two-pore domain potassium channel TREK-1 [4]. This background channel is regulated by a number of physiological stimuli [22]. In the heart, TREK-1 is believed to act as a stretch sensor, which possibly modulates atrial natriuretic peptide (ANP) secretion [23]. *Trek-1* null mutants in mice have a normal lifespan, and no obvious morphological or physiological pathology in the heart was reported [24]. Using *Xenopus* oocytes as a heterologous expression system, TREK-1 has been identified as a specific interaction partner of Popdc1 [4]. Co-expression of both Popdc1 and TREK-1 results in a current, which is approximately 2-fold higher than without Popdc1 and probably is caused by an increased membrane presence of TREK-1. It is therefore plausible that Popdc proteins modulate TREK-1 trafficking. Importantly, in the presence of theophylline, which increases cAMP levels, the effect of Popdc co-expression is abolished and no increase in TREK-1 conductivity was observed [4]. Based on the fact that TREK-1 current is enhanced in the presence of Popdc1, the null mutant should have a lower level of membrane-localized TREK-1. Since TREK-1 acts as a background channel setting the resting membrane potential, a reduction of TREK-1 current should lead to a situation in which myocytes would be more excitable, however the opposite is observed in the null mutant. Therefore it is likely that also the trafficking of other ion channels should depend on the interaction with Popdc proteins and defining these interactions might give a clue why *Popdc1 and -2* mutants develop a stress-induced bradycardia phenotype.

### 4.2 Caveolin-3

Caveolae are invaginations of the plasma membrane, which are involved in compartmentalization and cluster formation of membrane proteins and thereby improving signal transduction [25]. Important protein components of caveolae are cavins and caveolins [26]. Caveolin-3 (Cav-3) is the muscle-specific isoform, which localizes to the sarcolemma in skeletal muscle fibers and in the plasma membrane and t-tubules in cardiac myocytes [27]. Caveolae play various physiological roles, e.g., in vesicular trafficking, mechanosensation and transduction, and in signaling processes such as β- adrenergic signaling, and therefore in the control of cAMP production [28]. A number of different ion channels and transporters have been localized to caveolae in cardiac myocytes including LTCC, SCN5A, HCN4, NCX1, and others [29].

Cav-3 has recently been identified as an interaction partner of Popdc1 [19]. In the absence of *Popdc1*, caveolae in cardiac myocytes were altered in number and size. These alterations might contribute to the observed ischaemia/reperfusion vulnerability of *Popdc1*-null mutant hearts, but may also explain in part the cardiac arrhythmia phenotypes in *Popdc1* and *-2* mutants [19]. In this regard, it is noteworthy that Cav-3 was also found to be an interaction partner for Ca_v_1.2 and that caveolae in cardiac myocytes foster cluster formation of Ca_v_1.2 and the β2-AR, PKA and AC, thus allowing compartmentalized cAMP production and signal transduction [28]. It is therefore possible that Popdc proteins, being an interaction partner of Cav-3, are part of this signaling complex. In support of this notion it was found that isolated cardiac myocytes of *Popdc1* mutants display a reduction in [Ca^2+^]_i_ transients [19].

## 5 The Other cAMP Effector Proteins

### 5.1 Protein Kinase A (PKA)

PKA is the main effector of cAMP signaling. In its inactive form PKA is a tetramer composed of two regulatory (R) and two catalytic (C) subunits. There are four genes encoding regulatory subunits (RIα, RIβ, RIIα and RIIβ) and three genes encoding catalytic subunits (Cα, Cβ and Cγ). In the absence of cAMP, a dimer of C subunits are bound and suppressed by two R subunits [30]. cAMP binds to the R subunits causing a conformational change that relieves the inhibitory effect on the C subunits allowing phosphorylation of PKA targets. In the heart, PKA regulates excitation-contraction coupling through the phosphorylation of a number of key proteins such as troponin I, ryanodine receptor (RyR), phospholamban (PLB) and L-type calcium channels (LTCCs) causing an increase in the force (inotropy) and frequency (chronotropy) of the heartbeat and an increase in cardiac relaxation (lusitropy) [31]. SAN pacemaking is triggered by the rhythmic release of calcium from the sarcoplasmatic reticulum (SR) via the RyR, which is coupled to sarcolemmal currents capable of generating periodic action potentials [32]. SAN cells have high basal levels of cAMP and display elevated PKA-dependent phosphorylation of phospholamban (PLB) compared with other cardiac cell types [33]. In addition, PKA also triggers changes in gene expression through phosphorylation of nuclear target proteins such as CREB and HDAC4 and -5 [34–36]. Activation of PKA is compartmentalized due to recruitment of PKA to scaffolding proteins (AKAP), which anchor PKA close to its phosphorylation targets [37]. Apart from PKA, AKAP proteins bind AC, PDE, protein phosphatases, GPCRs and even other cAMP effector proteins and therefore generate macromolecular signaling complexes [38].

### 5.2 The Exchange Factor Directly Activated by cAMP (EPAC)

The Exchange Factor Directly Activated by cAMP (Epac) is encoded by two distinct genes *Rapgef3* (Epac1) and *Rapgef4* (Epac2) [39]. EPAC proteins are multi-domain proteins, which contain an N-terminal regulatory and a C-terminal catalytic region [40,41]. The regulatory region contains a high-affinity cAMP-binding domain, while the catalytic region entails a putative guanine nucleotide exchange factor (GEF) domain for both Rap1 and Rap2. Studies in neonatal rat cardiac myocytes show that an EPAC-selective cAMP agonist (8-pCPT-2'-O-Me-cAMP, 8-CPT) induces an increase in spontaneous Ca^2+^-oscillations [42], while in the adult mouse heart ventricular arrhythmia are triggered [43]. The 8-CPT-induced increase in Ca^2+^ spark frequency is caused by Ca^2+^/calmodulin-dependent protein kinase II (CaMKII)-dependent phosphorylation of the RyR [44]. Interestingly, the SR Ca^2+^-leak is triggered by EPAC-2 activation, suggesting isoform-specific roles in myocytes [45]. EPAC activation also enhances cell-cell coupling between myocytes through gap junction formation [46]. Another aspect of EPAC function is its role in cardiac hypertrophy. 8-CPT treatment or forced expression of EPAC-1 causes an increase in cell surface area, protein synthesis, and ANF expression and these effects were reversed by shRNA-mediated knockdown of Epac1 [47]. Hypertrophy-inducing Epac1 signaling involves small GTPases, phospholipase C (PLC), calcineurin and CaMKII, which activate nuclear factor of activated T-cells (NFAT) and myocyte enhancer factor 2 (MEF2) [48].

### 5.3 The Hyperpolarization-Activated Cyclic Nucleotide-Gated (HCN) Channels

The HCN channels are essential components of cardiac pacemaking in sinuatrial cells and the encoded proteins are responsible for a cation current known as I_f_ [49]. A unique property of I_f_ is the activation at a hyperpolarized membrane potential and it is therefore thought that I_f_ contributes to the pacemaker potential [49]. However there is an emerging consensus that a network of ion channels and ion pumps at the plasma membrane and in the sarcoplasmatic reticulum act together to generate the oscillating pacemaker action potential [50].

Of the four HCN channels present in the mammalian genome, three channels (HCN1, HCN2 and HCN4) are expressed in the heart [51]. While mutant mice for *HCN2* have a relatively mild sinus dysrhythmia [52], both *HCN1* and *HCN4* null mutants display defective cardiac pacemaking [53–55]. Loss of *HCN4* causes embryonic lethality due to a severe bradycardia [56], while the *HCN1* null mutant has a postnatal sinus bradycardia [54]. Consistent with the role of HCN channels in cardiac pacemaking is the observation that the I_f_ antagonist ivabradine, which binds HCN channels in its closed state [57], reduces heart rate in heart failure patients [58]. Modeling and biophysical measurements have clearly established that cAMP-binding enhances channel opening [59] and therefore this property would be the ideal mechanism for triggering the chronotropic response after adrenergic stimulation. However, a number of reports in the literature demonstrate that pacemaking in adult mice harboring cAMP-binding site mutations in *HCN4* retain the ability to respond to sympathetic stimulation, while embryonic hearts can only accelerate heart rate in the presence of a functional cAMP-binding site in HCN4 [60,61]. Data in patients either support [62], or refuse a role of If in the autonomous control of pacemaking [63]. There is also evidence that HCN4 is a PKA substrate and that inhibition of PKA significantly reduces the ability of beta-adrenergic agonists to shift the voltage dependence of I_f_ in isolated sinuatrial myocytes [64]. Thus, it is gradually emerging that different elements of the cAMP signal transduction pathway in sinuatrial myocytes act in parallel to mediate the adrenergic control of cardiac pacemaking.

## 6 Conclusions

The Popdc gene family is associated with cAMP signaling and therefore is part of the network of cAMP effector proteins. HCN channels and Popdc proteins display tissue-specific expression in the heart, while the other two cAMP effector proteins are more widely expressed. In particular the various elements of the cardiac conduction system display high-level expression of HCN1 and HCN4 [51] and Popdc1 and Popdc2 [4]. Thus, adrenergic control of the cardiac pacemaking and conduction may in particular be regulated via HCN channels and Popdc proteins. However, both PKA and EPAC also have their inputs in particular by modulating calcium release from the SR through the RyR2 [39] and calcium uptake through phosphorylation of PLB [33].

We probably need to consider that the evaluation of gene function by knockout approaches alone has its limitation when confronted with a complex network of protein families with redundant and overlapping functions. In case of Popdc proteins, we have, on the one hand, functional redundancy due to the presence of three Popdc proteins with overlapping expression pattern and similar if not identical functions [4]. In addition, however, there is also overlap in function at the level of the different cAMP effector proteins. Thus chronotropism of the cardiac pacemaker may involve the simultaneous activation of all four cAMP-binding proteins and therefore the loss of a single gene in one of the cAMP effector classes is compensated to a large extent by the remaining protein network.

However, if this assumption is correct, why vertebrates have maintained the same number of Popdc genes? An accidental loss of a gene encoding one of the mediator genes would be largely compensated and should be without severe consequences. A possible answer to this question might be that the heart has a vital role for a successful completion of the fight or flight response. Possibly, the speed with which the system can respond is key to the specific roles of Popdc proteins. Speed is undoubtedly an essential determinant of success in the fight or flight response. Since Popdc proteins have a high affinity for cAMP and are abundantly present in cardiac cells makes them ideally suited to mediate a rapid response to rising cAMP levels [4].

Thus in addition to the characterization of different animal models with a gain- and loss-of-Popdc gene function, it will be important to identify also small molecules, which are specific to the different cAMP effector proteins. These will assist in determining the specific functions of each cAMP effector protein. Such agonists and antagonists already exists for PKA [65], EPAC [66] and were recently discovered also for HCN channels [67]. Such small molecules represent important tools for advancing our knowledge of the different functions of the various cAMP effector proteins and would in particular advance our knowledge of the function of Popdc proteins in the heart.
